# Metagenomic Approach for Understanding Microbial Population from Petroleum Muck

**DOI:** 10.1128/genomeA.00533-14

**Published:** 2014-05-29

**Authors:** M. N. Joshi, S. V. Dhebar, S. V. Dhebar, P. Bhargava, A. S. Pandit, R. P. Patel, A. K. Saxena, S. B. Bagatharia

**Affiliations:** Gujarat State Biotechnology Mission, Department of Science & Technology, Government of Gujarat, Udyog Bhavan, Gandhinagar, Gujarat, India

## Abstract

Petroleum products play a major role in fueling the economy of the world but the pollution they create has become a critical issue. Understanding the diversity present in pipeline muck will help with the exploration of new microbial strains with better hydrocarbon degrading capacities for bioremediation of polluted sites. This study provides an analysis of petroleum muck using next generation sequencing.

## GENOME ANNOUNCEMENT

Petroleum is considered to be a principal source of energy and petroleum products are used in a wide variety of industries such as agriculture, plastics, tires, pharmaceuticals, dyes, detergents, and others. However, the accidental release of petroleum leads to widespread pollution of soil and aquifers, stimulating the need for an upgrade in bioremediation processes. Metagenomic studies of petroleum associated samples explore both culturable and unculturable microbial diversity that may play an important role in bioremediation of sites contaminated due to oil spills. Previously reported data describe the microbial diversity of oil contaminated sites using metagenomic approaches (1–3). However, no attempt has been made to study the microbial population of petroleum pipelines. Such sites assume greater importance than oil contaminated sites because pipelines will contain only microorganisms that are resistant or able to degrade hydrocarbons while an indigenous microbial population will also be present in oil contaminated sites. This study is aimed toward unravelling the taxonomic and functional diversity of microorganisms present in muck samples. The petroleum muck sample used here was kindly provided by the Indian Oil Corporation, Kandla.

The metagenomic study was based on next generation sequencing using the Ion Torrent platform. To our knowledge, this is the first report which describes a next generation sequencing based study of microbial diversity from petroleum pipelines. Metagenomic DNA extraction was carried out using the Power Soil DNA Isolation kit (MoBioLaboratories, Inc., Carlsbad, CA, USA). Sequencing was performed with a high-throughput Ion Torrent Personal Genome Machine with the Ion Torrent Server (Torrent Suite, version 3.2) using Ion Express Template 300 chemistry on a 318 chip, quality filtered, then exported in FastQ format. A total of 249 Mb data containing 2,228,423 sequences with an average length of 111 bps was obtained. Metagenomic reads were annotated with metagenome rapid annotation using the Subsystem Technology (MG-RAST) server (http://metagenomics.nmpdr.org/) (4). For contig level analysis, assembly was done with a MetaVelvet 1.13 assembler (5) using a max k-mer length of 51. Assemblies were uploaded to MG-RAST and the Integrated Microbial Genomes database (http://img.jgi.doe.gov/mer) (6). The taxonomic analysis revealed predominance of domain *Bacteria* (88.90%), followed by *Eukaryota* (0.06%) and *Archaea* (0.03%). Sequences affiliated with phylum *Proteobacteria* (99.09%) were most abundant, with *Gammaproteobacteria* (51.31%) as the major class and *Pseudomonas stutzeri* as the most abundant organism. *Pseudomonas stutzeri* strains are able to metabolize benzoate, cresol, naphthalene, xylene, toluene, and phenol (7). Other sequences belonged to phyla *Actinobacteria* (0.70%), *Firmicutes* (0.11%), and 0.75% other phyla. Although sequences were not affiliated with domain *Archaea* as much as other major phyla, *Euryarchaeota*, *Thaumarchaeota*, and *Crenarchaeota*, have developed mechanisms of metal resistance and thus can be used in bioremediation (8, 9).

This study will form a basis for the understanding of indigenous microbial populations existing in petroleum samples which will help in devising isolation strategies for culturable organisms and cloning of better enzymes that may be used in bioremediation of petroleum polluted sites.

### Nucleotide sequence accession number.

DNA sequences obtained have been deposited at NCBI Sequence Read Archive under the accession no. SRX314771.
